# Design of a Scaffold Parameter Selection System with Additive Manufacturing for a Biomedical Cell Culture

**DOI:** 10.3390/ma11081427

**Published:** 2018-08-14

**Authors:** Marc Rabionet, Emma Polonio, Antonio J. Guerra, Jessica Martin, Teresa Puig, Joaquim Ciurana

**Affiliations:** 1 Oncology Unit (TargetsLab), Department of Medical Sciences, Faculty of Medicine, University of Girona, Emili Grahit 77, 17003 Girona, Spain; m.rabionet@udg.edu (M.R.); emma.polonio@udg.edu (E.P.); jessica.martin@udg.edu (J.M.); 2 Department of Mechanical Engineering and Industrial Construction, University of Girona, Maria Aurèlia Capmany 61, 17003 Girona, Spain; antonio.guerra@udg.edu

**Keywords:** scaffold, PCL, RepRap, fused filament fabrication, three-dimensional, cell culture

## Abstract

Open-source 3D printers mean objects can be quickly and efficiently produced. However, design and fabrication parameters need to be optimized to set up the correct printing procedure; a procedure in which the characteristics of the printing materials selected for use can also influence the process. This work focuses on optimizing the printing process of the open-source 3D extruder machine RepRap, which is used to manufacture poly(ε-caprolactone) (PCL) scaffolds for cell culture applications. PCL is a biocompatible polymer that is free of toxic dye and has been used to fabricate scaffolds, i.e., solid structures suitable for 3D cancer cell cultures. Scaffold cell culture has been described as enhancing cancer stem cell (CSC) populations related to tumor chemoresistance and/or their recurrence after chemotherapy. A RepRap BCN3D+ printer and 3 mm PCL wire were used to fabricate circular scaffolds. Design and fabrication parameters were first determined with SolidWorks and Slic3r software and subsequently optimized following a novel sequential flowchart. In the flowchart described here, the parameters were gradually optimized step by step, by taking several measurable variables of the resulting scaffolds into consideration to guarantee high-quality printing. Three deposition angles (45°, 60° and 90°) were fabricated and tested. MCF-7 breast carcinoma cells and NIH/3T3 murine fibroblasts were used to assess scaffold adequacy for 3D cell cultures. The 60° scaffolds were found to be suitable for the purpose. Therefore, PCL scaffolds fabricated via the flowchart optimization with a RepRap 3D printer could be used for 3D cell cultures and may boost CSCs to study new therapeutic treatments for this malignant population. Moreover, the flowchart defined here could represent a standard procedure for non-engineers (i.e., mainly physicians) when manufacturing new culture systems is required.

## 1. Introduction

Scaffolds are solid structures usually made of a polymeric material that is used for a wide range of applications. They provide a necessary support for three-dimensional (3D) cell growth, thanks to their biocompatibility and biodegradability [1], and are extremely useful in in vitro 3D cell cultures. Traditional cell culture is applied to two-dimensional (2D) models on flat surfaces, but this methodology is not representative of the cells’ physiological environment and usually confers them with less malignancy. The literature has reported that 3D cell culture with scaffolds can increase the cancer stem cell (CSC) population [2,3,4]. CSCs correspond to a small population within the tumor which is resistant to chemotherapy and capable of dividing to form the tumor again after treatment (this is known as recurrence or metastasis) [5,6]. Since this malignant subpopulation represents a small percentage within the tumor, the population expansion and enrichment described would help in their study and promote further development of therapeutic strategies.

**Figure 1:**
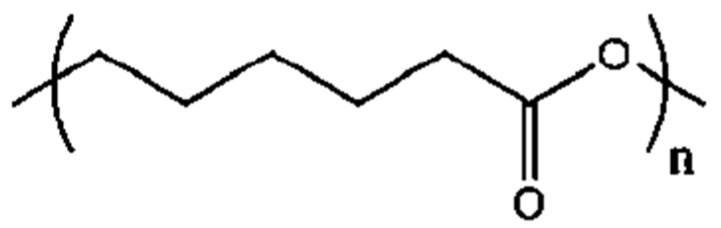
Poly(ε-caprolactone) chemical structure.

Additive manufacturing (AM) technologies have arisen as a novel set of tools with which to fabricate scaffolds [5,7]. In particular, 3D printers based on fused filament fabrication (FFF) technology are one of the most accessible and simplest options [8]. They are open-source, low-cost machines which usually use thermoplastic materials [9,10] and can easily be modified to improve the quality of the printed 3D products [11]. A variety of biocompatible polymers can be used for scaffold production with FFF. Poly-L-lactic acid (PLA) is a biodegradable thermoplastic aliphatic polyester that has great potential in clinics thanks to its biocompatibility and restorability. Consequently, it is widely used in tissue engineering [12]. Poly(ε-caprolactone) (PCL; Figure 1) is also a biodegradable polyester proven to be biocompatible and toxic-dye-free, but it has a slower degradation rate and different mechanical and physical features. For instance, PCL has a lower melting point (60 °C), reflecting its lower hydrogen bonding and polarity which determine its chemical and molecular behavior. Moreover, PCL does not have any isomers so there are no variances in the melting temperature and biological degradation. Due to these characteristics, its use in tissue engineering, drug delivery, and cell cultures is increasing [2,3,6,13,14]. PCL can be also used as copolymers, such as PCL-collagen and PCL-gelatin, and in combination with other polymers, for example PLA or PEG [13,15].

As scaffold production with 3D printers is a new area, greater effort should be made to determine the optimal parameters for the process [1,6,9]. The processing parameters in question are closely related to the properties of the polymer chosen and the subsequent application intended for the scaffold(s). First, the design parameters determine the architecture of the scaffold and can comprise the filament diameter, the distance between filaments, and the deposition angle [16]. They can also be modified depending on the desired design and application of the scaffold. Second, fabrication parameters control the printing process. These parameters include the extruder and bed temperature, deposition velocity, and layer height, and are closely linked to the material of the polymer and the environment [9,17].

When scaffolds are produced for tissue engineering or regenerative medicine, controlling features, such as pore size, pore shape, or mechanical strength, is mandatory [9,18]. Although there are some studies into the 3D printing of scaffolds based on fused deposition modeling (FDM) [19,20] very few analyze the effects the architecture of the scaffold may have on cell proliferation, and none develop schematic procedures or methods aimed at retaining any knowledge gained. Grémare et al., [21] studied the physicochemical and biological properties of PLA scaffolds produced by 3D printing (FFF). The authors studied four different square pore sizes (0, 150, 200, and 250 um). Results showed that scaffold pore size had negligible effects on their mechanical properties. After three and seven days of human bone marrow stromal cell (HBMSC) culture being applied, the scaffolds exhibited excellent viability and homogeneous distribution regardless of the pore size. Hutmancher et al. [22] studied the mechanical and cell culture response of PCL scaffolds using 61 ± 1% porosity and two matrix architectures. Results showed that five-angle scaffolds had significantly lower stiffness under compression loading than those with a three-angle pattern. Data also revealed that in terms of cell proliferation, while a scaffold with a 0/60/120° lay-down pattern had a higher proliferation rate in the first 2 weeks, the scaffolds with a 0/72/144/36/108° lay-down overtook the three-angle matrix architecture in Weeks 3 and 4. Recently, Rabionet et al. [23] analyzed the effects of tubular scaffold architecture on cell proliferation for vascular applications. Results showed the strong influence the 3D process parameters have on the scaffold architecture and, subsequently, cell proliferation. Narrow pores produced lower cell proliferation due to the lower oxygen and nutrient exchange. 

As the literature has reported, cell proliferation onto a scaffold depends on the material, the architecture, and cell kinetics. Whenever physicians need to work with cells, they require the best scaffolding features to obtain ideal cell culture results. In fact, the main problem was that scaffolds did not provide the same results for different lines of cells when the cells are cultured. When working with cells, physicians have different purposes and goals. For instance, they may want to enrich or treat the cells or to determine the impact a drug is having/has had on the cells. While identical scaffold features do not provide the same results, the cell line does. In fact, each cell line works better with different scaffold features. For this reason, this work aims to optimize the design features and the selection of the manufacturing process parameters when the open-source 3D extruder machine RepRap is utilized. This methodology focuses on manufacturing PCL scaffolds suitable for 3D cancer cell cultures and CSCs expansion as a first step before expanding to other cell lines. Both design and fabrication parameters have been optimized by following a specific flowchart step by step, and checking a measurable variable. In addition, preliminary in vitro experiments were performed to study the impact the scaffold design and fabrication have on the efficiency physicians require from the 3D cell culture and the scaffolds produced. Therefore, a sample application for the mass production of PCL scaffolds using a low-cost machine could be used to improve cancer stem cell research. The flowchart developed here provides a novel methodology to adjust process parameters to print micrometric scaffolds suitable for three-dimensional cell culture because, as is demonstrated, each cell line required different scaffold features. Hence, an optimization diagram could represent a common procedure which could be used by non-engineering professionals when a 3D cell culture protocol has to be established de novo. Physicians working with 3D cell cultures usually need some kind of rules or guidelines to follow to set up the cell culture. This paper’s contribution is the methodology required to set up the 3D printing technology for a new line of cell culture by first defining the design characteristics and then the parameter selection for the manufacturing process. This paper does not contribute to the knowledge about PCLs or the 3D printing machine itself, but instead provides a methodology for physicians. The contribution is the method and steps to follow when scaffolds need to be manufactured for a new cell line. 

## 2. Experimental Setup

### 2.1. Material

A 3 mm poly(ε-caprollactone) (PCL) wire (Perstorp, Malmö, Sweden) with a density of 1145 Kg/m^3^ and a molecular weight of 80,000 g/mol, was used to fabricate circular scaffolds 19 mm in diameter (Corning Life Sciences, New York, NY, USA). PCL is a biodegradable polyester with a low melting point (60 °C) and a glass transition of about −60 °C. 

### 2.2. Three-Dimensional Printer Machine

**Figure 2:**
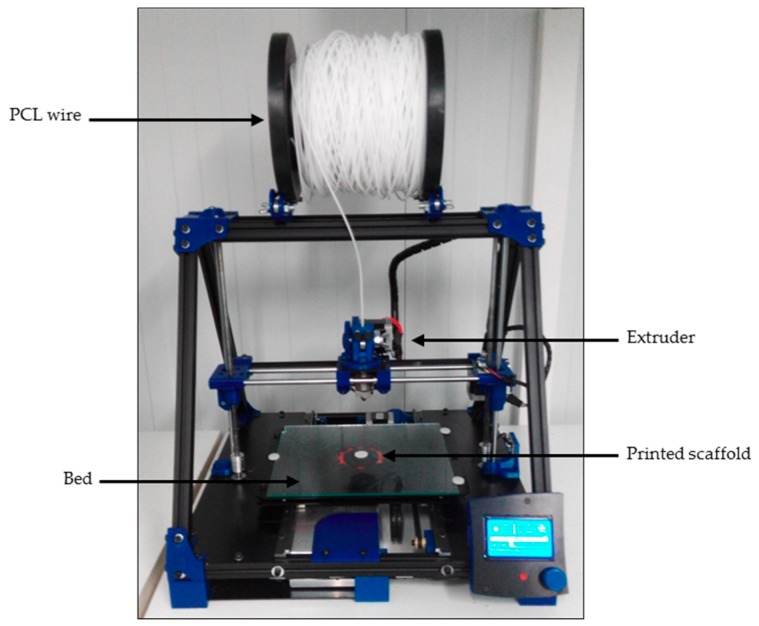
RepRap BCN 3D+ printer with a 3 mm PCL wire.

An open-source and modular RepRap BCN 3D+ printer (CIM, Barcelona, Spain) was used to produce three-dimensional scaffolds (Figure 2). This printer was selected because of its capacity to allow a user to optimize its parameters as they see fit. It uses fused filament fabrication (FFF). First, the filament was unwound from a roll of wire and supplied to the extruder. Then, the material was extruded through the nozzle using different temperatures depending on the value being tested. Finally, the printed filament was deposited onto a heated platform (also known as a bed).

### 2.3. Scaffold Design and Additive Manufacturing

SolidWorks (Waltham, Massachusetts, Estats Units) was the computer-aided design (CAD) software chosen for the scaffolds’ design. The stereolithography (STL) file formats the designs that were transferred to the computer-aided manufacturing (CAM) software Slic3r to establish the fabrication parameters. This software, while maintaining the SolidWorks design, generated G-code files which can control and regulate the machine to obtain the correctly-printed scaffolds. Scaffold design features were selected based on other research work focused on tissue engineering which had similar goals to this work, i.e., cell enrichment or treatment, or drug delivery applicability. The features are described in Table 1. Previous screening experiments were carried out to adjust the range of the scaffold design features.

### 2.4. Process Parameter Optimization

**Figure 3:**
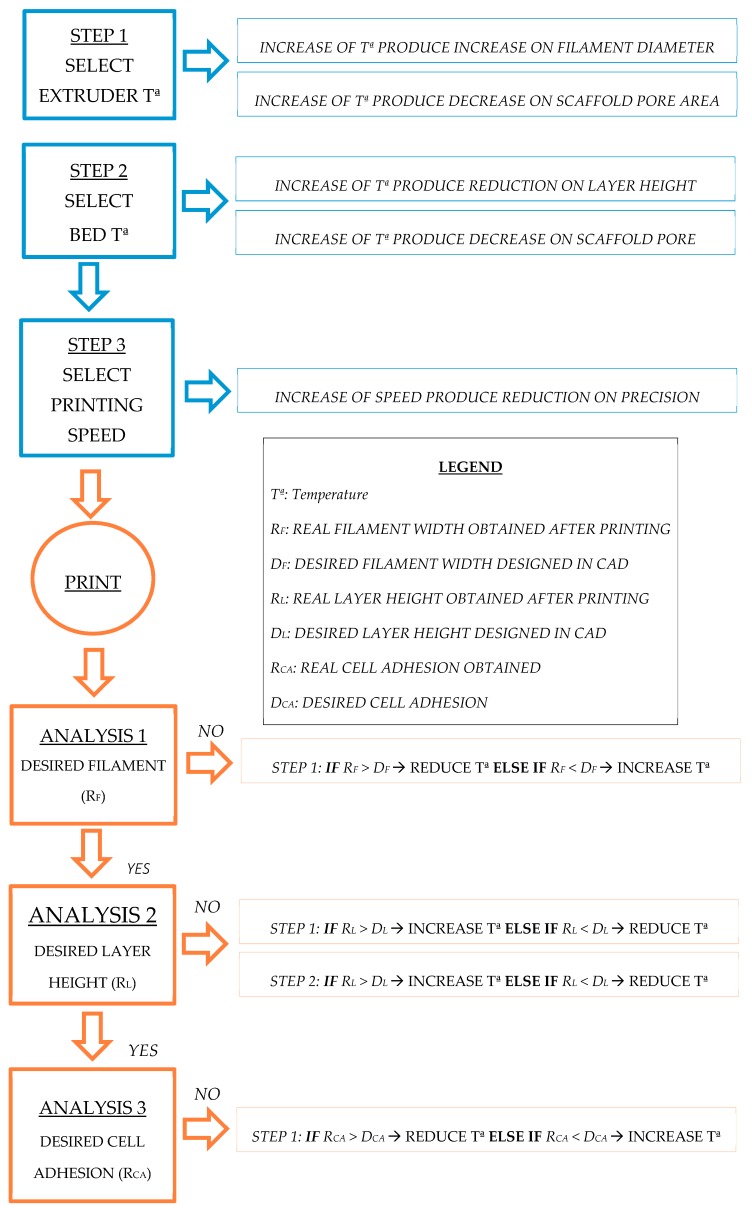
Flowchart of process parameter optimization. Every parameter consists of the values tested and, on the right, the corresponding measurable variable for new cell cultures. Fabrication parameters are in the left column and design parameters in the right.

The fabrication parameters and design feature values used for the experimental setup are shown in Table 1. A wide range of characteristics and parameter values were selected from the literature as the screening values with which to start. A wide range of processing parameters were selected based on the research work focused on tissue engineering with similar goals to ours, i.e., the enrichment or treatment of cells of the applicability for drug delivery. Thus, previous screening experiments were carried out to adjust the range of the processing parameters for the scaffolds. By following a sequential flowchart (Figure 3), the optimal tested value to be selected for each parameter was determined. Optimization was first performed using a generic geometrical form. A fixed circular scaffold design was used as the control pattern: 0.4 mm in diameter and layer height extruded filament, 1 mm distance between filaments, 90° deposition angle, 19 mm in diameter scaffold, and eight scaffold layers. As optimization progressed, design feature values were replaced by the optimal ones, resulting in a final scaffold design suitable for three-dimensional cancer cell culture. Furthermore, the cancer cell culture is now more like real physiological conditions, including an enrichment of the CSCs’ subpopulation. Each step on the flowchart presented in Figure 3 included parameter testing and a physical scaffold variable measurement to assess the quality of the printing. Thus, optimal parameter values were sequentially determined and considering the final application as the optimal function to be reached. Physical variables, such as printed filament diameter, first layer height, and real distance between filaments, were measured using an inverted optical microscope (Nikon, Tokyo, Japan). Printed structures, as well as a nanometric ruler, were placed on the stage. Binomial variables (material adhesion, adhesion of contiguous filaments, printing quality such as the absence of blobs etc.) were assessed by sight. Finally, the cell efficiency of the different deposition angles was evaluated through a three-dimensional breast cancer cell culture to validate the parameters selected. Breast CSCs were used because their expansion would represent a new opportunity to develop new treatments against cancer stem features related to cancer relapse and metastasis.

### 2.5. Cell Line

MCF-7 breast carcinoma cells (ATCC^®^ HTB-22™) and NIH/3T3 murine fibroblasts cell lines (ATCC^®^ CRL-1658™) were obtained from the American Type Culture Collection (ATCC, Rockville, MD, USA). MCF-7 and NIH/3T3 cells were cultured in DMEM (Dulbecco’s Modified Eagle’s Medium) (Gibco, Walthman, MA, USA) supplemented with 10% fetal bovine serum, 1% l-glutamine (which means 2 mM l-glutamine), 1% sodium pyruvate (which means 1 mM sodium pyruvate), 50 U/mL penicillin and 50 µg/mL streptomycin (HyClone, Logan, UT, USA). Cells were maintained at 37 °C and in a 5% CO_2_ atmosphere.

### 2.6. Scaffold Sterilization

Scaffolds were sterilized following a previously-described methodology [2,24]. Meshes were submerged in a 70% ethanol/water solution overnight, washed with PBS (Gibco, Walthman, MA, USA), and finally exposed to UV light for 30 min. Only the top side was irradiated because PCL has a semi-transparent behavior when exposed to UV wavelengths [25]. This sterilization method was followed to avoid any changes in the stents’ final properties [18].

### 2.7. Three-Dimensional Cell Culture in Scaffolds

Scaffolds were designed by considering their subsequent use in regular 12-well cell culture microplates. First, cells were detached from the original cell culture microplate and counted using the trypan blue dye method. As viable cells possess an intact membrane, trypan blue cannot penetrate them, but as dead cells have an altered membrane the dye can penetrate them. Therefore, trypan blue was added in a cell sample and cell viability was counted using a Neubauber Chamber (Marienfeld-Superior, Lauda-Königshofen, Germany) and an inverted optical microscope. A total of 100,000 cells (MCF-7) or 40,000 (NIH/3T3) in 250 µL cell suspension were placed onto the center of the scaffolds’ surface to allow cell attachment. After 3 h of incubation, 1.5 mL of fresh medium was added to cover the scaffold and the cells were incubated for 72 h. Then, the scaffold was placed in a new well to quantify only the cells attached. It was washed with PBS and 1 mL of trypsin was added. After incubation, 2.5 mL of fresh medium was added, and the cell suspension was collected and centrifuged at 1500 rpm for 5 min. Finally, the supernatant was discarded, and the cells were re-suspended and counted.

### 2.8. Statistical Analysis

Results were collected from at least six independent experiments. All data are expressed as mean ± standard error (SE). Data were analyzed by Student’s *t* test.

## 3. Results: Scaffolds Production

Following the method developed, experimental work was first carried out to find the best way to produce scaffolds which can sustain cell cultures. Sequential work was done to set scaffold design features and manufacturing process parameters.

### 3.1. Optimization of Process Parameters

Processing parameters were optimized to achieve high quality scaffold printing for cell culture application. Thus, different physical scaffold variables were measured to ensure the correct fit between the computer design and the printed scaffold. The processing parameters included both fabrication and design parameters as shown in the “Experimental Setup” section (Table 1). Processing parameters were chosen according to the literature and the state-of-art [9,11,16,17]. However, the process optimization methodology explained here, based on a sequential flowchart (Figure 3), is both innovative and unique.

Experiments were initially carried out with a generic scaffold design (see Section 2.4 “Methods”) to set the fabrication parameters and then adjusted to the design parameters required to produce the scaffolds.

Fabrication parameters (extruder and bed temperature, deposition velocity, and layer height) were introduced with Slic3r software. These parameters are related to the characteristics of the polymeric material (mainly PCL) and the printing process. However, different values were tested for the parameters (by checking the measurable variable mentioned in Table 1) in order to meet scaffold manufacturing requirements.

Once the polymeric material and its fabrication parameters had been characterized and set, design features were subsequently established using the SolidWorks 3D software. Parameters, such as filament diameter, distance between filaments, and deposition angle, were tested. These are related to the three-dimensional design of the scaffold and the effect they have on the cancer cell culture.

First, to determine the optimal fabrication parameters, a fixed scaffold design was established as a control pattern: 90° deposition angle, 0.4 mm in diameter filament and 1 mm distance between filaments. This enabled us to do printings with the same design, but different fabrication parameters, to find the optimal ones. Later, as the design parameters were optimized, they were replaced.

Following the flowchart defined in Figure 3, all the parameters were characterized and selected sequentially to obtain the appropriate setup for producing 3D-printed scaffolds. The optimization of each process parameter is described in the following sections.

### 3.2. Extruder Temperature

Poly(ε-caprolactone) was chosen as the polymer to work with because of its compatibility with cell cultures. PCL has a low melting point (60 °C). To achieve enough malleability and considering there is some heat dissipation, higher temperatures were also tested to find the optimal value (Table 1). A fixed scaffold design described in the Methods section was printed. Then, *the printed filament diameter* was measured as a physical variable. Low extruder temperatures (65–80 °C) could not melt the material enough, thus the amount of the extruded material was low. As a consequence, the printed filament diameter was smaller than the one designed (0.4 mm). High temperatures (90 °C) melt the polymer excessively and also increase the diameter of the filament due to flattening and some blobs being produced. Therefore, the optimal extruder temperature was established at 85 °C. The printed filament diameter was 0.39 ± 0.05 mm.

### 3.3. Bed Temperature

To set the optimal bed temperature, a generic geometrical scaffold design was printed, and two different measurable variables were evaluated. Material adhesion was assessed as a binomial variable (yes/no), firstly testing the lowest temperature (25 °C, Table 1). If the printed material had not adhered enough to the surface (no), another printing was performed, this time with a higher bed temperature. Once the material had adhered to the surface (yes), the first layer height was then measured. Bed temperatures ranging from 25 to 33 °C gave a non-adherent first layer scaffold. In addition, much higher temperatures (37 °C) melt the material excessively, flattening the filament and decreasing the height of the first layer (lower than the 0.4 mm designed one). A 35 °C bed temperature was considered optimal as this allowed first layer adhesion and the filaments were not flattened. Their first layer height was 0.37 ± 0.07 mm.

### 3.4. Deposition Velocity

The goal with this parameter was to find a high deposition velocity without forgetting the quality of the printed scaffold. The printed filament diameter was chosen as the tangible variable with which to analyze the impact this parameter has on the scaffold. The optimal deposition velocity was established as being 10 mm/s. The filament diameter was 0.42 ± 0.05 mm. When the speed was faster (20 and 30 mm/s) the material did not have enough time to deposit itself on the surface, resulting in smaller filament diameter or sometimes even discontinuous filament production.

### 3.5. Filament Diameter

At this point, the diameter of the printed filament deposited on the collector was analyzed. Extrusion and deposition velocity can exert a direct influence on fiber morphology. Therefore, once he manufacturing velocity had been fixed, the diameter of the extruded filament was evaluated next. Three different design filament diameters were tested: 0.175, 0.3, and 0.5 mm. To ensure the filaments remained tangent along the vertical axis, the printer’s layer height was adjusted to each design filament diameter. Diameters that were too large caused the adhesion of two contiguous filaments, favored by their proximity and elevated temperature. For this reason, the first variable studied was the possible adhesion of contiguous filaments, such as a binomial variable (yes/no). Thus, only the values that did not cause the adhesion of two filaments in the same layer were selected to continue the analysis (0.175 and 0.3 mm). The second measured variable was the printed filament diameter. A design diameter of 0.175 mm caused erratic printing because the amount of material was too low to form a linear filament. The final value tested, 0.3 mm, was found to be optimal as it gave a printed filament diameter of 0.31 ± 0.02 mm. The established filament diameter value also determined the thickness of each layer. The scaffolds were manufactured with eight layers, so the final thickness of the scaffolds was 2.4 mm.

### 3.6. Layer Height

Layer height is defined as the distance between two connected layers along the *Z* axis. Since all layers are designed and printed on top of each other, this parameter was determined by the printed filament diameter. For this reason, the filament diameter, although being a design parameter, was established before finishing, optimizing the fabrication parameters (Figure 3). In some cases, the deposited material tends to flatten out and so the printed height is lower. At that point, two different values were analyzed: 0.3 mm (the whole filament diameter) and 0.28 mm (because of a certain flattening) and the quality of the printing recorded (absence of blobs). In this case, 0.3 mm was found to be the optimal layer height for our design as flattening, due to high temperatures, did not occur. When evaluating smaller established heights, the printing process produced blobs.

The absence of filament flattening may be attributed to the relatively low extruder temperature used, (85 °C, see Section 2.1) which can be considered low compared with other biocompatible polymers used in 3D printing, such as PLA [9,12].

### 3.7. Distance between Filaments

This is a key parameter because it affects the pore size of the scaffold [9]. This design parameter consists of the shortest distance between the axis of two filaments located within the same layer. We were interested in achieving small pore sizes, thus, we focused on the testing small distances (0.5, 0.7, 1 mm). Nevertheless, small distances between filaments may be problematic if two contiguous filaments join. For this reason, the real distance between filaments was measured to take into account whether this value matched that of the one expected (designed). 

**Figure 4:**
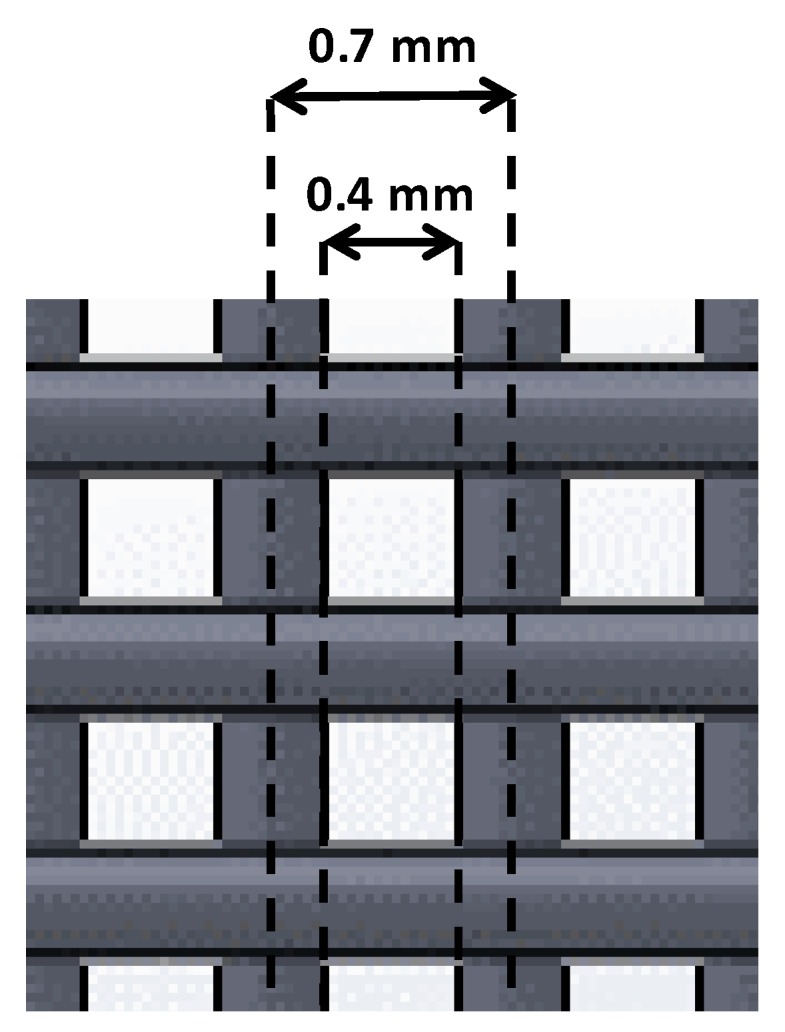
Distances between two contiguous filaments. Axis (0.7 mm) and outer distance (0.4 mm) are represented. Filament diameter was fixed at 0.3 mm.

Distances of 0.7 and 1 mm gave no filament joining, so real distances were higher than 0. Within these values, the smallest value was chosen (0.7 mm). Taking into account this parameter and the optimal filament diameter previously established, the distance between the outer parts of two contiguous filaments was, consequently, 0.4 mm (Figure 4).

### 3.8. Deposition Angle 

Once all previous parameters were optimized, three different scaffolds with different deposition angles were designed and manufactured, thus obtaining different pore characteristics, which may influence cell attachment and growth (Table 2). As high-quality printings for all three designs were achieved, it was agreed to test the adequacy for 3D cell culture with all three designs.

**Figure 5:**
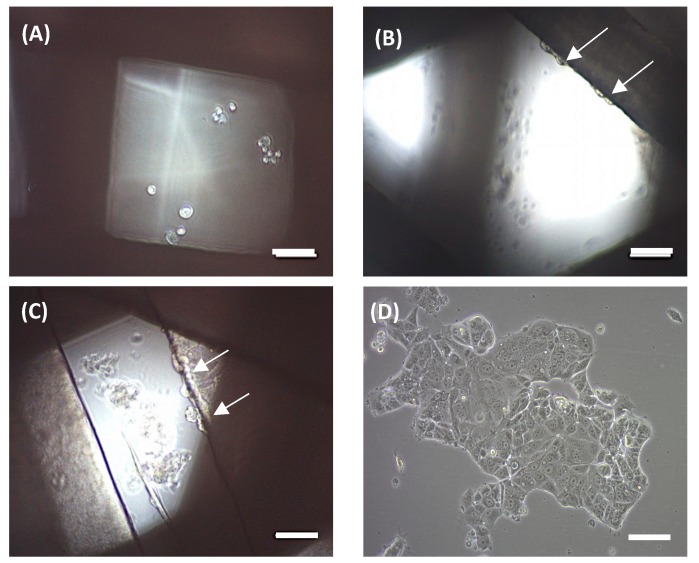
Optical microscope images of MCF-7 cells seeded on the scaffolds. In 90° scaffolds, cells were attached at the bottom of the well (**A**). In 45° and 60° scaffolds, cells were attached both on the scaffold and at the well (**B**,**C**, respectively). (**D**) MCF-7 cells in 2D culture. White arrows on the images indicate cells adhered to PCL filaments. Scale bars represent 100 µm.

An MCF-7 breast carcinoma cell line was used to preliminarily evaluate scaffold ability in terms of three-dimensional cell culture. MCF-7 cells were seeded onto scaffolds and cultivated for 72 h. Then, attached cells were trypsinized and counted. No cells were counted on the 90° scaffolds. Under an optical microscope, no cells were observed on the filament, but rather attached at the bottom of the microplate well (Figure 5a), which is in agreement with cell counting. Scaffolds of 45° showed a subtle cell adhesion of 3.52 ± 1.16% when compared with the 2D control. We subsequently tested 60° scaffolds, which showed an increased cell adhesion of 26.50 ± 10.98%. In both cases, cells were previously observed at the well bottom and attached to the scaffold filaments, with the last ones are indicated by white arrows (Figure 5b,c, respectively).

**Figure 6:**
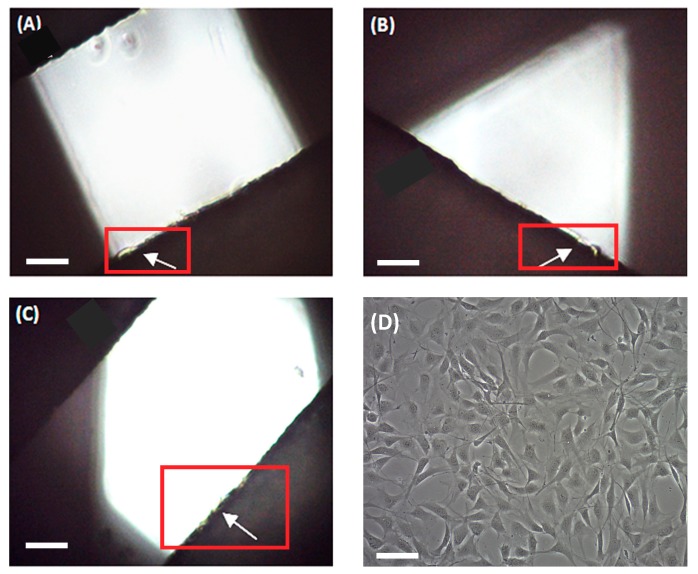
Optical microscope images of NIH/3T3 cells seeded on the scaffolds. Cells were attached on scaffolds of 90° (**A**), 60° (**B**), and 45° (**C**). (**D**) NIH/3T3 cells in a 2D culture. White arrows on the images indicate cells adhered to PCL filaments. Scale bars represent 100 µm.

Then, scaffolds were also evaluated through fibroblast cell cultures. Murine NIH/3T3 fibroblasts were seeded onto the three designs during 72 h and cell proliferation was assessed. In this case, fibroblasts adhered to all three scaffold models (Figure 6), with the highest cell proliferation value being found on the 90° design (56.30 ± 5.03% compared to the 2D control). The other two architectures exhibited slightly smaller values. For instance, 60° scaffolds presented a 49.52 ± 5.62% cell growth and 45° models, 39.11 ± 8.12%, compared to the monolayer culture.

### 3.9. Optimal Process Parameters Values

After the optimization experiments and basic cell culture tests had been completed, the optimal processing parameters for PCL scaffold printing were established (see Table 3) once the methodology had been applied to set each optimal parameter for cell cultures and for future experiments with CSCs culture enrichment using PCL scaffolds.

## 4. Discussion

In this work, a methodology to optimize the processing parameters for PCL scaffold production using a RepRap 3D printer has been developed. By using an optimization flowchart, PCL scaffolds suitable for cell culture were manufactured (Figure 3). The optimal processing parameters determined are closely related to those defined in other studies using the same technology and material. Domingos et al., (2013) set up an 80 °C printing temperature, 10 mm/s velocity, an approximately 0.3 mm filament diameter and a layer height of 0.28 mm [15]. A previous study by the same research group used an extrusion temperature of 70 °C and a speed of 8 mm/s [1]. These small variations support the idea of using a single, common methodology (Figure 3) to optimize the processing parameters. Compared with previous work in the literature, the flowchart provided here makes it easier to adjust scaffold design features and processing parameters according to cell line characteristics. Several case studies were run to validate the flowchart depicted in Figure 3. Results show how cell culture is improved by using scaffolds which allow cell cultures to be created in 3D conditions and optimized based on the cells’ features. In addition, process parameters were also evaluated using cell culture experiments. All scaffold culture experiments presented sterility resulting from the sterilization procedure described here. Both 60° and 45° scaffolds showed adequate design parameters for the MCF-7 cell cultures. In particular, the 60° scaffold design displayed the highest percentage of cell attachment, and exhibited good biocompatibility for the MCF-7 breast cancer cells. In contrast, the NIH/3T3 fibroblast cells presented a more homogeneous growth along the three scaffold designs. However, the 90° scaffold showed the highest cell proliferation value. Therefore, different kinds of cells may prefer different scaffold architectures, further demonstrating the need of a common procedure to find the optimal values. Moreover, a tumor and a non-tumor cell line were tested, showing the flexibility of the flowchart described here.

Three-dimensional cell culture on scaffolds may also be improved by other fabrication-independent parameters such as polarity of cell culture plates, culture media and time [26], as well as different cell culture types, including a dynamic model [27]. This optimization will be the focus of further studies as we attempt to improve cell attachment percentages. Furthermore, CSC population enrichment by cell culture on scaffolds will be evaluated using different approaches. 

To date, most of the work related to scaffold production focuses on optimizing design features and forgets about improving fabrication parameters [1,9,16]. In this work, a flowchart to optimize the parameters of the whole process has been proposed (Figure 3) to help with their selection. In addition, this methodology may be further used to set up scaffold manufacturing (both the design features and the fabrication parameters) when using a RepRap 3D printer or any other AM technologies and/or materials.

## 5. Conclusions

In this work, the design features and fabrication parameters of scaffolds and the RepRap 3D printer were optimized to produce PCL scaffolds suitable for three-dimensional cell cultures. The optimization was performed following a detailed and unidirectional flowchart, thus providing some procedural guidelines with great potential for other popular manufacturing technologies and materials. The contribution of this paper is for scaffolds made with PCL materials. However, this experiment was only carried out to validate the methodology developed as a valuable method for future cell cultures. Often, physicians work with 2D cell cultures, but, as seen here, 3D cell cultures appear to be good method of improving cell culture enrichment. Furthermore, as the design features and manufacturing parameters need to be set for the different cell lines used each time, this methodology will help physicians and other operators to do just that.

Moreover, the scaffolds produced were proven to allow cell attachment and cell growth. The 60° scaffold design mainly worked for the MCF-7 cells and the 90° for the NIH/3T3 fibroblasts. Three-dimensional cell cultures with PCL scaffolds fabricated with a 3D printer offer both researchers and clinics a set of novel applications for the future. The flowchart developed represents a new tool with which to quickly manufacture scaffolds for a wide range of applications, including cell cultures and tissue engineering. For instance, the use of 3D cell cultures can boost CSC populations to study new therapeutic treatment. 

## Figures and Tables

**Table 1:** Process parameters used for PCL scaffold printing.

	Parameters	Tested Values	Measurable Variable
Fabrication parameters	Extruder temperature	65, 70, 75, 80, 85, and 90 °C	Printed filament diameter
	Bed temperature	25, 30, 33, 35, and 37 °C	1-Material adhesion (Y/N) 2-First layer height
	Deposition velocity	10, 20, and 30 mm/s	Printed filament diameter
	Layer height	0.28 and 0.3 mm	Printing quality (absence of blobs)
Design parameters	Filament diameter	0.175, 0.3, and 0.5 mm	1-Adhesion of contiguous filaments (Y/N) 2-Printed filament diameter
	Distance between filaments	0.5, 0.7, and 1 mm	1-Real distance between filaments 2-Smallest pore option
	Deposition angle	90°, 45°, and 60°	Pore angles

**Table 2:** Scaffold designs with different deposition angles: 90°, 45°, and 60°.

Deposition Angles	Pore Shape	Area	Plan View
90°	Square	0.16 mm^2^	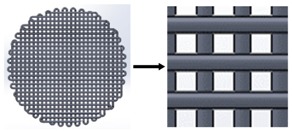
45°	Six variable forms (triangles and irregular polygons)	1.98 × 10^−4^ to 0.13 mm^2^	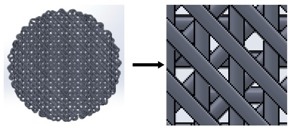
60°	Equilateral triangle	0.1256 mm^2^	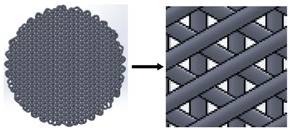

**Table 3:** Optimal processing parameter values to be used for PCL scaffold printing.

Title	Parameters	Optimal Values
Manufacturing parameters	Extruder temperature	85 °C
	Bed temperature	35 °C
	Deposition velocity	10 mm/s
	Layer height	0.3 mm
Design parameters	Filament diameter	0.3 mm
	Distance between filaments	0.7 mm
	Deposition angle	60° (MCF-7 breast cancer cells) 90° (NIH/3T3 murine fibroblasts)
